# *N*,*N*-Diethyl-2-hy­droxy­ethanaminium 5-(2,4-di­nitro­phen­yl)barbiturate sesquihydrate

**DOI:** 10.1107/S1600536813012257

**Published:** 2013-05-11

**Authors:** Govindan Mangaiyarkarasi, Doraisamyraja Kalaivani

**Affiliations:** a PG and Research Department of Chemistry, Seethalakshmi Ramaswami College, Tiruchirappalli 620 002, Tamil Nadu, India

## Abstract

In the title hydrated mol­ecular salt, C_6_H_16_NO^+^·C_10_H_5_N_4_O_7_^−^·1.5H_2_O [systematic name: *N*,*N*-diethyl-2-hy­droxy­ethan­am­in­ium 5-(2,4-di­nitro­phen­yl)-2,6-di­oxo-1,2,3,6-tetra­hydro­pyrim­idin-4-olate sesquihydrate], the dihedral angle between the six-membered rings in the anion is 37.66 (11)°. The nitro groups *ortho* and *para* to the ring junction are rotated from their attached ring by 40.8 (3) and 23.5 (3)°, respectively. The ethanol group is disordered over two of the ‘arms’ of the cation in a statistical ratio. In the crystal, [010] chains of anions occur, linked by N—H⋯O and O—H⋯O hydrogen bonds, which generate *R*_2_^2^(8) loops. Further N—H⋯O and O—H⋯O hydrogen bonds link the components into a three-dimensional network. One of the water O atoms lies near an inversion centre and is 50% occupied.

## Related literature

For related barbiturates, see: Kalaivani *et al.* (2008 ▶); Kalaivani & Buvaneswari (2010 ▶); Buvaneswari & Kalaivani (2011 ▶); Babykala & Kalaivani (2012 ▶).
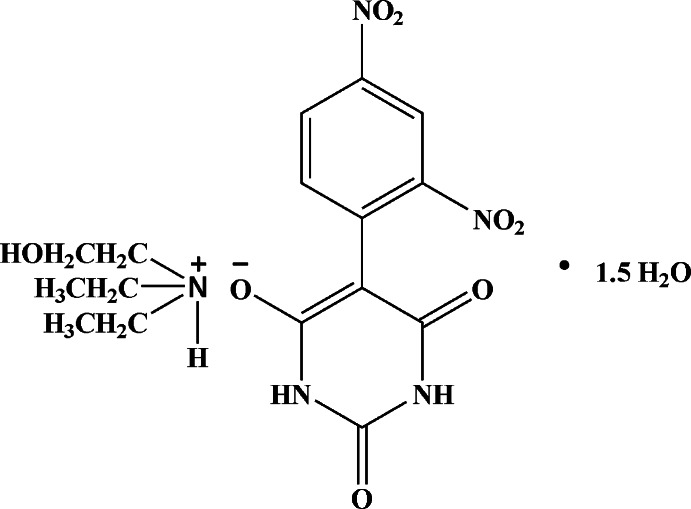


## Experimental

### 

#### Crystal data

C_6_H_16_NO^+^·C_10_H_5_N_4_O_7_^−^·1.5H_2_O
*M* *_r_* = 438.40
Monoclinic, 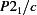

*a* = 9.6003 (5) Å
*b* = 11.6568 (6) Å
*c* = 18.1993 (9) Å
β = 98.066 (3)°
*V* = 2016.51 (18) Å^3^
*Z* = 4
Mo *K*α radiation
μ = 0.12 mm^−1^
*T* = 293 K
0.30 × 0.20 × 0.20 mm

#### Data collection

Bruker Kappa APEXII CCD diffractometer
Absorption correction: multi-scan (*SADABS*; Bruker, 2004) ▶ *T*_min_ = 0.948, *T*_max_ = 0.979
16207 measured reflections
3219 independent reflections
2353 reflections with *I* > 2σ(*I*)
*R*_int_ = 0.033

#### Refinement

*R*[*F* ^2^ > 2σ(*F* ^2^)] = 0.043
*wR*(*F* ^2^) = 0.131
*S* = 1.09
3219 reflections
341 parameters
49 restraints
H atoms treated by a mixture of independent and constrained refinement
Δρ_max_ = 0.34 e Å^−3^
Δρ_min_ = −0.26 e Å^−3^

### 

Data collection: *APEX2* (Bruker, 2004 ▶); cell refinement: *SAINT* (Bruker, 2004 ▶); data reduction: *SAINT*; program(s) used to solve structure: *SIR92* (Altomare *et al.*, 1993 ▶); program(s) used to refine structure: *SHELXL97* (Sheldrick, 2008 ▶); molecular graphics: *ORTEP-3 for Windows* (Farrugia, 2012 ▶) and *Mercury* (Macrae *et al.*, 2008 ▶); software used to prepare material for publication: *SHELXL97*.

## Supplementary Material

Crystal structure: contains datablock(s) global, I. DOI: 10.1107/S1600536813012257/hb7074sup1.cif

Structure factors: contains datablock(s) I. DOI: 10.1107/S1600536813012257/hb7074Isup2.hkl

Supplementary material file. DOI: 10.1107/S1600536813012257/hb7074Isup3.cml

Additional supplementary materials:  crystallographic information; 3D view; checkCIF report

## Figures and Tables

**Table 1:** Hydrogen-bond geometry (Å, °)

*D*—H⋯*A*	*D*—H	H⋯*A*	*D*⋯*A*	*D*—H⋯*A*
O8—H8⋯O9^i^	0.82	2.14	2.739 (10)	130
O9—H9⋯O11^ii^	0.82	2.11	2.841 (17)	148
N3—H3⋯O3^iii^	0.86	1.94	2.794 (2)	174
N4—H4*A*⋯O1^iv^	0.86	1.97	2.804 (3)	165
N5—H5*A*⋯O10^ii^	0.97 (4)	1.86 (4)	2.808 (4)	164 (3)
O10—H10*B*⋯O2	0.90 (2)	1.87 (3)	2.751 (3)	163 (5)
O10—H10*A*⋯O6^iv^	0.89 (2)	2.02 (2)	2.902 (4)	172 (6)

Symmetry codes: (i) 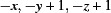; (ii) 
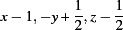
; (iii) 
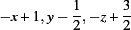
; (iv) 
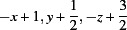
.

**Fig. 1.**
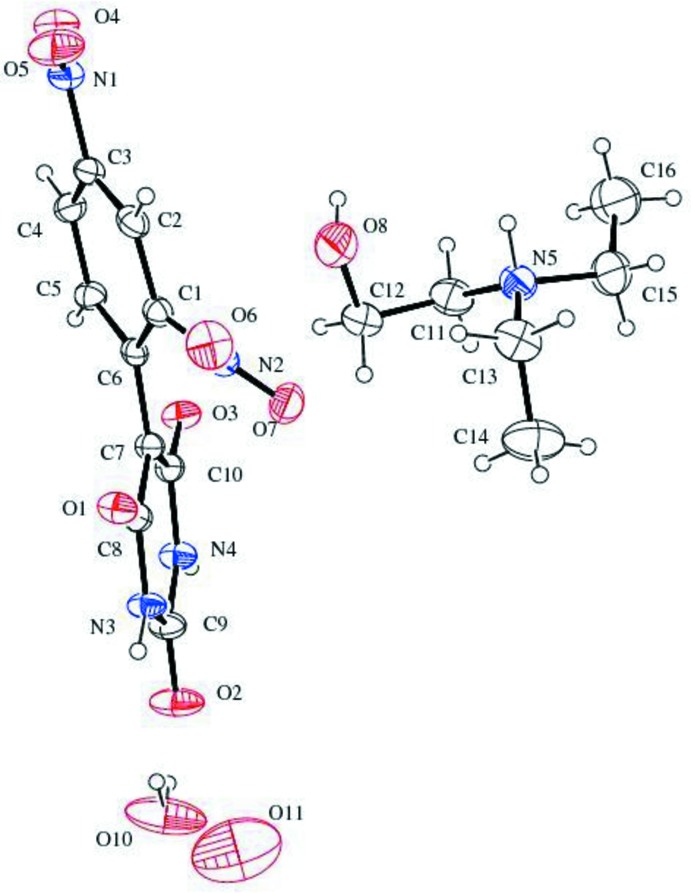
The ORTEP diagram of the title compound with 30% probability displacement ellipsoids.

**Fig. 2.**
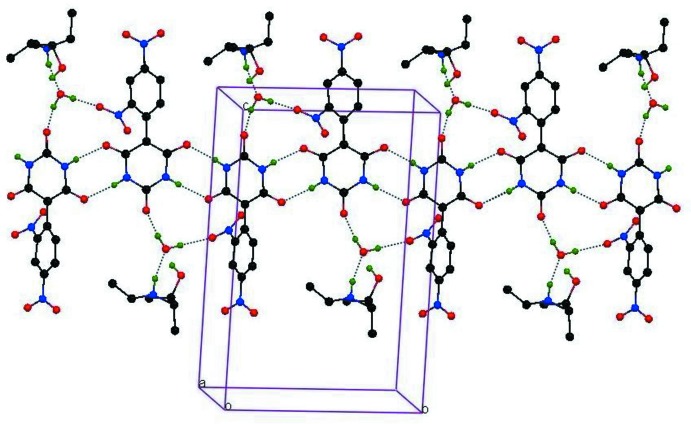
Packing view of title compound.

## supplementary crystallographic information

### Comment

Good crystallinity of the title barbiturate (pyrimidine derivative) and the biological effects of the related barbiturates (Kalaivani *et al.*,2008; Kalaivani & Buvaneswari,2010) necessitate us to subject the molecular salt of the present investigation for single-crystal X-ray analysis. The crystal structures of two different barbiturates with *N*,*N*-diethyl-2-hydroxyethanaminium cation have been reported by us recently (Buvaneswari & Kalaivani,2011; Babykala & Kalaivani,2012). No disorder has been observed in their cation moieties. The X-ray results of the molecular salt of present interest imply that the asymmetric unit consists of *N*,*N*-Diethyl-2-hydroxyethanaminium cation, 5-(2,4-dinitrophenyl)barbiturate anion and 1.5 molecules of water (Scheme).Fig.1 is the *ORTEP* diagram of the title molecule with 30% probability displacement ellipsoids. Disorder has been noticed in the ethanol group of diethylethanolammonium cation at two positions with equal probability (50% occupancy). The crystal structure features a number of N—H···O and O—H···O hydrogen bonds (Table.1) and these hydrogen bond interactions extend three dimensionally (Fig.2). O11 of water molecule is also disordered with its symmetry related position (*sym*:-*x* + 1,-*y*,-*z* + 2).The hydrogen atoms of O11 could not be located.In the anion, the dinitrophenyl ring and the barbiturate ring are not planar and the dihedral angle between them is 37.65 (0.09)°.The nitro groups attached to C3 and C1 atoms of phenyl ring make dihedral angles,23.51(0.15°) and 39.75(0.11°) respectively with the phenylplane. The inversion related barbiturate residues are linked through *R*^2^_2_(8) motifs.

### Experimental

1-Chloro-2,4-dinitrobenzene(2.02 g, 0.01 mol) was dissolved in 20 ml of ethanol and mixed with barbituric acid(1.2 g, 0.01 mol) dissolved in 30 ml of ethanol. To this mixture diethylethanolamine (5.85 g, 0.05 mol) was added,the red coloured solution obtained was shaken well for 3 h and kept as such at 25° C. Dark shiny maroon red crystals were deposited from the solution after 4 weeks. The crystals were washed with 5 ml of ethanol followed by 30 ml of ether, powdered well and was further washed with 40 ml of dry ether to remove unreacted reactants.The pure crystals thus obtained were recrystallized from hot ethanol(yield:85%; m.pt; 511 K). Red blocks were obtained by slow evaporation of an ethanol solution at room temperature.

### Refinement

The initial refinement of the structure showed abnormal displacement ellipsoids for two chains of diethylethanolammonium ion, soon it could be identified as disorder arising from the ethanol and ethyl group exchanging positions. The disordered components are suitably positioned and the occupancies refined, keeping the sum of occupancies as 1. As the occupancies convulsed to 50%, their values were fixed as 0.5 towards the last cycles of refinement.

### Crystal data

**Table:**

C_6_H_16_NO^+^·C_10_H_5_N_4_O_7_^−^·1.5H_2_O	*F*(000) = 924
*M**_r_* = 438.40	*D*_x_ = 1.444 Mg m^−^^3^
Monoclinic, *P*2_1_/*c*	Mo *K*α radiation, λ = 0.71073 Å
*a* = 9.6003 (5) Å	Cell parameters from 5746 reflections
*b* = 11.6568 (6) Å	θ = 2.1–24.1°
*c* = 18.1993 (9) Å	µ = 0.12 mm^−^^1^
β = 98.066 (3)°	*T* = 293 K
*V* = 2016.51 (18) Å^3^	Block, red
*Z* = 4	0.30 × 0.20 × 0.20 mm

### Data collection

**Table:**

Bruker Kappa APEXII CCD diffractometer	3219 independent reflections
Radiation source: fine-focus sealed tube	2353 reflections with *I* > 2σ(*I*)
Graphite monochromator	*R*_int_ = 0.033
ω and φ scan	θ_max_ = 24.2°, θ_min_ = 2.1°
Absorption correction: multi-scan (*SADABS*; Bruker, 2004)	*h* = −11→10
*T*_min_ = 0.948, *T*_max_ = 0.979	*k* = −13→13
16207 measured reflections	*l* = −20→20

### Refinement

**Table:**

Refinement on *F*^2^	Secondary atom site location: difference Fourier map
Least-squares matrix: full	Hydrogen site location: inferred from neighbouring sites
*R*[*F*^2^ > 2σ(*F*^2^)] = 0.043	H atoms treated by a mixture of independent and constrained refinement
*wR*(*F*^2^) = 0.131	*w* = 1/[σ^2^(*F*_o_^2^) + (0.0518*P*)^2^ + 1.3181*P*] where *P* = (*F*_o_^2^ + 2*F*_c_^2^)/3
*S* = 1.09	(Δ/σ)_max_ = 0.001
3219 reflections	Δρ_max_ = 0.34 e Å^−^^3^
341 parameters	Δρ_min_ = −0.26 e Å^−^^3^
49 restraints	Extinction correction: *SHELXL97* (Sheldrick, 2008), Fc^*^=kFc[1+0.001xFc^2^λ^3^/sin(2θ)]^-1/4^
Primary atom site location: structure-invariant direct methods	Extinction coefficient: 0.0070 (12)

### Special details

**Table:**

Geometry. All e.s.d.'s (except the e.s.d. in the dihedral angle between two l.s. planes) are estimated using the full covariance matrix. The cell e.s.d.'s are taken into account individually in the estimation of e.s.d.'s in distances, angles and torsion angles; correlations between e.s.d.'s in cell parameters are only used when they are defined by crystal symmetry. An approximate (isotropic) treatment of cell e.s.d.'s is used for estimating e.s.d.'s involving l.s. planes.
Refinement. Refinement of *F*^2^ against ALL reflections. The weighted *R*-factor *wR* and goodness of fit *S* are based on *F*^2^, conventional *R*-factors *R* are based on *F*, with *F* set to zero for negative *F*^2^. The threshold expression of *F*^2^ > σ(*F*^2^) is used only for calculating *R*-factors(gt) *etc*. and is not relevant to the choice of reflections for refinement. *R*-factors based on *F*^2^ are statistically about twice as large as those based on *F*, and *R*- factors based on ALL data will be even larger.

### Fractional atomic coordinates and isotropic or equivalent isotropic displacement parameters (Å^2^)

**Table:**

	*x*	*y*	*z*	*U*_iso_*/*U*_eq_	Occ. (<1)
C1	0.2628 (2)	0.0366 (2)	0.54157 (13)	0.0329 (6)	
C2	0.2250 (3)	0.0267 (2)	0.46611 (13)	0.0391 (6)	
H2	0.1497	−0.0193	0.4465	0.047*	
C3	0.3010 (3)	0.0862 (2)	0.42078 (13)	0.0382 (6)	
C4	0.4145 (3)	0.1532 (2)	0.44884 (13)	0.0392 (6)	
H4	0.4629	0.1959	0.4174	0.047*	
C5	0.4544 (3)	0.1556 (2)	0.52411 (13)	0.0356 (6)	
H5	0.5339	0.1975	0.5429	0.043*	
C6	0.3811 (2)	0.09788 (18)	0.57400 (12)	0.0302 (5)	
C7	0.4339 (2)	0.09933 (19)	0.65315 (12)	0.0307 (5)	
C8	0.4299 (2)	−0.0016 (2)	0.69591 (12)	0.0320 (5)	
C9	0.5512 (3)	0.0971 (2)	0.80407 (13)	0.0415 (6)	
C10	0.4982 (2)	0.1991 (2)	0.68671 (12)	0.0333 (6)	
C11	0.055 (3)	0.421 (2)	0.6323 (12)	0.123 (6)	0.50
H11A	0.0931	0.4283	0.6844	0.148*	0.50
H11B	0.0582	0.4964	0.6100	0.148*	0.50
C12	0.148 (2)	0.341 (2)	0.5957 (12)	0.123 (6)	0.50
H12A	0.2171	0.3879	0.5757	0.148*	0.50
H12B	0.1987	0.2941	0.6346	0.148*	0.50
O8	0.0978 (6)	0.2768 (6)	0.5468 (3)	0.0968 (18)	0.50
H8	0.0804	0.3126	0.5078	0.145*	0.50
C11'	0.0599 (11)	0.4204 (8)	0.6337 (5)	0.0340 (19)	0.50
H11C	0.0687	0.4851	0.6012	0.041*	0.50
H11D	0.0916	0.4447	0.6842	0.041*	0.50
C12'	0.1500 (14)	0.3260 (12)	0.6142 (7)	0.058 (2)	0.50
H12C	0.1463	0.2636	0.6483	0.087*	0.50
H12D	0.2452	0.3526	0.6169	0.087*	0.50
H12E	0.1173	0.3002	0.5647	0.087*	0.50
C13	−0.1176 (4)	0.2726 (3)	0.66133 (19)	0.0675 (9)	
H13A	−0.0693	0.2132	0.6374	0.081*	
H13B	−0.2176	0.2562	0.6517	0.081*	
C14	−0.0709 (5)	0.2668 (4)	0.7436 (2)	0.0950 (13)	
H14A	0.0297	0.2732	0.7535	0.142*	
H14B	−0.0996	0.1948	0.7624	0.142*	
H14C	−0.1130	0.3285	0.7676	0.142*	
C15	−0.169 (2)	0.4779 (19)	0.6581 (13)	0.125 (6)	0.50
H15A	−0.1266	0.4882	0.7093	0.149*	0.50
H15B	−0.2639	0.4505	0.6590	0.149*	0.50
C16	−0.179 (3)	0.5919 (19)	0.6233 (11)	0.120 (6)	0.50
H16A	−0.2529	0.6364	0.6412	0.144*	0.50
H16B	−0.0910	0.6330	0.6347	0.144*	0.50
O9	−0.2118 (8)	0.5734 (6)	0.5442 (3)	0.109 (2)	0.50
H9	−0.2877	0.5405	0.5351	0.164*	0.50
C15'	−0.1878 (13)	0.4742 (14)	0.6532 (12)	0.073 (4)	0.50
H15C	−0.2805	0.4412	0.6536	0.088*	0.50
H15D	−0.1517	0.4977	0.7034	0.088*	0.50
C16'	−0.198 (2)	0.5752 (15)	0.6036 (11)	0.104 (6)	0.50
H16C	−0.1060	0.5972	0.5945	0.156*	0.50
H16D	−0.2409	0.6376	0.6267	0.156*	0.50
H16E	−0.2550	0.5563	0.5575	0.156*	0.50
N1	0.2581 (2)	0.0802 (2)	0.34106 (12)	0.0495 (6)	
N2	0.1609 (2)	−0.01230 (19)	0.58625 (12)	0.0427 (5)	
N3	0.4909 (2)	0.00326 (16)	0.76952 (10)	0.0392 (5)	
H3	0.4902	−0.0585	0.7953	0.047*	
N4	0.5523 (2)	0.19167 (17)	0.76131 (10)	0.0397 (5)	
H4A	0.5898	0.2526	0.7822	0.048*	
N5	−0.0911 (3)	0.3855 (2)	0.62698 (15)	0.0531 (6)	
O1	0.37759 (18)	−0.09499 (13)	0.67247 (9)	0.0400 (4)	
O2	0.5996 (3)	0.09640 (17)	0.87033 (10)	0.0673 (7)	
O3	0.50875 (19)	0.29377 (14)	0.65590 (9)	0.0432 (5)	
O4	0.2933 (2)	0.1579 (2)	0.30267 (10)	0.0642 (6)	
O5	0.1890 (3)	−0.0026 (2)	0.31603 (11)	0.0756 (7)	
O6	0.1012 (2)	−0.10192 (18)	0.56472 (12)	0.0613 (6)	
O7	0.1327 (2)	0.04142 (17)	0.63940 (11)	0.0542 (5)	
O10	0.7928 (4)	0.1814 (3)	0.98317 (18)	0.1345 (15)	
O11	0.5047 (15)	−0.0413 (8)	0.9782 (6)	0.193 (5)	0.50
H5A	−0.126 (4)	0.376 (3)	0.574 (2)	0.079 (10)*	
H10B	0.744 (5)	0.145 (4)	0.944 (2)	0.16 (2)*	
H10A	0.818 (6)	0.251 (3)	0.969 (3)	0.19 (3)*	

### Atomic displacement parameters (Å^2^)

**Table:**

	*U*^11^	*U*^22^	*U*^33^	*U*^12^	*U*^13^	*U*^23^
C1	0.0335 (13)	0.0302 (13)	0.0342 (13)	0.0035 (11)	0.0017 (10)	−0.0016 (10)
C2	0.0345 (13)	0.0388 (14)	0.0408 (14)	0.0048 (11)	−0.0057 (11)	−0.0061 (11)
C3	0.0418 (14)	0.0416 (15)	0.0289 (13)	0.0114 (12)	−0.0029 (11)	−0.0019 (11)
C4	0.0476 (15)	0.0373 (14)	0.0327 (14)	0.0037 (12)	0.0061 (12)	0.0011 (11)
C5	0.0407 (14)	0.0325 (13)	0.0325 (13)	−0.0009 (11)	0.0009 (11)	−0.0012 (11)
C6	0.0355 (13)	0.0228 (12)	0.0313 (12)	0.0077 (10)	0.0013 (10)	−0.0021 (10)
C7	0.0376 (13)	0.0249 (12)	0.0286 (12)	0.0022 (10)	0.0015 (10)	−0.0013 (10)
C8	0.0364 (13)	0.0295 (13)	0.0295 (12)	0.0028 (11)	0.0024 (10)	−0.0023 (10)
C9	0.0589 (17)	0.0327 (14)	0.0303 (14)	0.0006 (13)	−0.0032 (12)	−0.0019 (11)
C10	0.0402 (14)	0.0280 (13)	0.0314 (13)	0.0021 (11)	0.0042 (11)	−0.0022 (10)
C11	0.117 (12)	0.123 (13)	0.123 (13)	−0.008 (10)	−0.003 (10)	0.002 (10)
C12	0.108 (11)	0.119 (12)	0.136 (12)	0.011 (8)	−0.005 (9)	−0.008 (9)
O8	0.089 (4)	0.126 (5)	0.073 (3)	0.028 (3)	0.004 (3)	−0.028 (3)
C11'	0.035 (4)	0.034 (4)	0.030 (4)	−0.007 (4)	−0.003 (3)	0.000 (3)
C12'	0.039 (4)	0.069 (6)	0.062 (5)	0.011 (4)	−0.004 (4)	0.002 (4)
C13	0.066 (2)	0.058 (2)	0.075 (2)	−0.0106 (16)	−0.0018 (17)	0.0037 (17)
C14	0.111 (3)	0.090 (3)	0.077 (3)	−0.028 (2)	−0.008 (2)	0.026 (2)
C15	0.193 (13)	0.101 (11)	0.076 (9)	0.043 (10)	0.007 (9)	−0.014 (8)
C16	0.189 (12)	0.085 (10)	0.079 (7)	0.042 (8)	−0.002 (7)	−0.018 (6)
O9	0.161 (7)	0.092 (4)	0.070 (4)	0.037 (4)	−0.001 (4)	0.002 (4)
C15'	0.055 (5)	0.061 (7)	0.110 (11)	0.024 (5)	0.037 (5)	−0.004 (7)
C16'	0.104 (8)	0.058 (7)	0.151 (17)	0.039 (6)	0.019 (10)	−0.007 (10)
N1	0.0520 (14)	0.0595 (16)	0.0343 (13)	0.0112 (13)	−0.0036 (11)	−0.0041 (12)
N2	0.0382 (12)	0.0411 (13)	0.0472 (13)	0.0017 (11)	0.0006 (10)	0.0016 (11)
N3	0.0602 (14)	0.0265 (11)	0.0284 (11)	−0.0010 (10)	−0.0029 (9)	0.0030 (9)
N4	0.0573 (14)	0.0272 (11)	0.0312 (11)	−0.0047 (10)	−0.0051 (10)	−0.0048 (9)
N5	0.0562 (16)	0.0510 (15)	0.0500 (15)	0.0065 (12)	0.0001 (12)	−0.0067 (12)
O1	0.0543 (11)	0.0248 (9)	0.0376 (10)	−0.0029 (8)	−0.0053 (8)	−0.0010 (7)
O2	0.1120 (18)	0.0475 (12)	0.0334 (11)	−0.0130 (12)	−0.0207 (11)	0.0022 (9)
O3	0.0687 (12)	0.0263 (9)	0.0327 (9)	−0.0060 (8)	0.0009 (8)	0.0006 (7)
O4	0.0747 (15)	0.0790 (16)	0.0362 (11)	0.0055 (12)	−0.0016 (10)	0.0101 (11)
O5	0.0957 (18)	0.0804 (16)	0.0435 (12)	−0.0150 (14)	−0.0150 (11)	−0.0126 (11)
O6	0.0528 (12)	0.0529 (13)	0.0769 (15)	−0.0191 (10)	0.0051 (10)	−0.0075 (11)
O7	0.0551 (12)	0.0581 (13)	0.0524 (12)	0.0048 (10)	0.0185 (9)	−0.0030 (10)
O10	0.173 (3)	0.106 (2)	0.097 (2)	−0.064 (2)	−0.076 (2)	0.0379 (19)
O11	0.262 (11)	0.162 (11)	0.153 (10)	−0.096 (10)	0.021 (9)	0.020 (6)

### Geometric parameters (Å, º)

**Table:**

C1—C2	1.375 (3)	C12'—H12D	0.9600
C1—C6	1.400 (3)	C12'—H12E	0.9600
C1—N2	1.472 (3)	C13—N5	1.495 (4)
C2—C3	1.366 (4)	C13—C14	1.504 (5)
C2—H2	0.9300	C13—H13A	0.9700
C3—C4	1.379 (4)	C13—H13B	0.9700
C3—N1	1.453 (3)	C14—H14A	0.9600
C4—C5	1.370 (3)	C14—H14B	0.9600
C4—H4	0.9300	C14—H14C	0.9600
C5—C6	1.397 (3)	C15—N5	1.47 (2)
C5—H5	0.9300	C15—C16	1.470 (13)
C6—C7	1.458 (3)	C15—H15A	0.9700
C7—C8	1.414 (3)	C15—H15B	0.9700
C7—C10	1.415 (3)	C16—O9	1.44 (2)
C8—O1	1.248 (3)	C16—H16A	0.9700
C8—N3	1.386 (3)	C16—H16B	0.9700
C9—O2	1.230 (3)	O9—H9	0.8200
C9—N4	1.350 (3)	C15'—C16'	1.478 (12)
C9—N3	1.351 (3)	C15'—N5	1.512 (15)
C10—O3	1.248 (3)	C15'—H15C	0.9700
C10—N4	1.387 (3)	C15'—H15D	0.9700
C11—N5	1.45 (2)	C16'—H16C	0.9600
C11—C12	1.509 (13)	C16'—H16D	0.9600
C11—H11A	0.9700	C16'—H16E	0.9600
C11—H11B	0.9700	N1—O4	1.220 (3)
C12—O8	1.21 (2)	N1—O5	1.223 (3)
C12—H12A	0.9700	N2—O7	1.214 (3)
C12—H12B	0.9700	N2—O6	1.229 (3)
O8—H8	0.8200	N3—H3	0.8600
C11'—C12'	1.474 (10)	N4—H4A	0.8600
C11'—N5	1.493 (11)	N5—H5A	0.97 (4)
C11'—H11C	0.9700	O10—H10B	0.903 (19)
C11'—H11D	0.9700	O10—H10A	0.89 (2)
C12'—H12C	0.9600	O11—O11^i^	1.260 (18)
			
C2—C1—C6	123.3 (2)	H13A—C13—H13B	107.6
C2—C1—N2	114.6 (2)	C13—C14—H14A	109.5
C6—C1—N2	121.8 (2)	C13—C14—H14B	109.5
C3—C2—C1	118.2 (2)	H14A—C14—H14B	109.5
C3—C2—H2	120.9	C13—C14—H14C	109.5
C1—C2—H2	120.9	H14A—C14—H14C	109.5
C2—C3—C4	121.7 (2)	H14B—C14—H14C	109.5
C2—C3—N1	118.5 (2)	N5—C15—C16	120.3 (17)
C4—C3—N1	119.7 (2)	N5—C15—H15A	107.2
C5—C4—C3	118.5 (2)	C16—C15—H15A	107.2
C5—C4—H4	120.8	N5—C15—H15B	107.2
C3—C4—H4	120.8	C16—C15—H15B	107.2
C4—C5—C6	123.0 (2)	H15A—C15—H15B	106.9
C4—C5—H5	118.5	O9—C16—C15	106.7 (16)
C6—C5—H5	118.5	O9—C16—H16A	110.4
C5—C6—C1	115.1 (2)	C15—C16—H16A	110.4
C5—C6—C7	120.0 (2)	O9—C16—H16B	110.4
C1—C6—C7	124.8 (2)	C15—C16—H16B	110.4
C8—C7—C10	119.4 (2)	H16A—C16—H16B	108.6
C8—C7—C6	120.1 (2)	C16—O9—H9	109.5
C10—C7—C6	120.4 (2)	C16'—C15'—N5	110.1 (12)
O1—C8—N3	117.5 (2)	C16'—C15'—H15C	109.6
O1—C8—C7	125.3 (2)	N5—C15'—H15C	109.6
N3—C8—C7	117.2 (2)	C16'—C15'—H15D	109.6
O2—C9—N4	122.5 (2)	N5—C15'—H15D	109.6
O2—C9—N3	122.0 (2)	H15C—C15'—H15D	108.2
N4—C9—N3	115.5 (2)	C15'—C16'—H16C	109.5
O3—C10—N4	116.9 (2)	C15'—C16'—H16D	109.5
O3—C10—C7	126.2 (2)	H16C—C16'—H16D	109.5
N4—C10—C7	116.9 (2)	C15'—C16'—H16E	109.5
N5—C11—C12	114.7 (18)	H16C—C16'—H16E	109.5
N5—C11—H11A	108.6	H16D—C16'—H16E	109.5
C12—C11—H11A	108.6	O4—N1—O5	123.5 (2)
N5—C11—H11B	108.6	O4—N1—C3	118.3 (2)
C12—C11—H11B	108.6	O5—N1—C3	118.2 (2)
H11A—C11—H11B	107.6	O7—N2—O6	123.1 (2)
O8—C12—C11	121 (2)	O7—N2—C1	118.7 (2)
O8—C12—H12A	107.2	O6—N2—C1	118.0 (2)
C11—C12—H12A	107.2	C9—N3—C8	125.4 (2)
O8—C12—H12B	107.2	C9—N3—H3	117.3
C11—C12—H12B	107.2	C8—N3—H3	117.3
H12A—C12—H12B	106.8	C9—N4—C10	125.7 (2)
C12—O8—H8	109.5	C9—N4—H4A	117.2
C12'—C11'—N5	111.9 (9)	C10—N4—H4A	117.2
C12'—C11'—H11C	109.2	C11—N5—C15	107.6 (13)
N5—C11'—H11C	109.2	C11—N5—C11'	1.2 (13)
C12'—C11'—H11D	109.2	C15—N5—C11'	108.0 (10)
N5—C11'—H11D	109.2	C11—N5—C13	116.3 (10)
H11C—C11'—H11D	107.9	C15—N5—C13	111.0 (9)
C11'—C12'—H12C	109.5	C11'—N5—C13	115.2 (4)
C11'—C12'—H12D	109.5	C11—N5—C15'	114.4 (11)
H12C—C12'—H12D	109.5	C15—N5—C15'	7.4 (15)
C11'—C12'—H12E	109.5	C11'—N5—C15'	114.9 (7)
H12C—C12'—H12E	109.5	C13—N5—C15'	108.8 (6)
H12D—C12'—H12E	109.5	C11—N5—H5A	107 (2)
N5—C13—C14	114.3 (3)	C15—N5—H5A	110 (2)
N5—C13—H13A	108.7	C11'—N5—H5A	108 (2)
C14—C13—H13A	108.7	C13—N5—H5A	105 (2)
N5—C13—H13B	108.7	C15'—N5—H5A	104 (2)
C14—C13—H13B	108.7	H10B—O10—H10A	109 (3)
			
C6—C1—C2—C3	−4.7 (4)	C6—C1—N2—O7	37.2 (3)
N2—C1—C2—C3	169.2 (2)	C2—C1—N2—O6	38.1 (3)
C1—C2—C3—C4	1.2 (4)	C6—C1—N2—O6	−147.8 (2)
C1—C2—C3—N1	−177.4 (2)	O2—C9—N3—C8	177.7 (3)
C2—C3—C4—C5	2.7 (4)	N4—C9—N3—C8	−1.1 (4)
N1—C3—C4—C5	−178.7 (2)	O1—C8—N3—C9	−179.5 (2)
C3—C4—C5—C6	−3.4 (4)	C7—C8—N3—C9	1.3 (4)
C4—C5—C6—C1	0.1 (3)	O2—C9—N4—C10	−179.1 (3)
C4—C5—C6—C7	176.8 (2)	N3—C9—N4—C10	−0.3 (4)
C2—C1—C6—C5	4.1 (3)	O3—C10—N4—C9	−179.9 (2)
N2—C1—C6—C5	−169.4 (2)	C7—C10—N4—C9	1.4 (4)
C2—C1—C6—C7	−172.5 (2)	C12—C11—N5—C15	−174.2 (17)
N2—C1—C6—C7	14.1 (3)	C12—C11—N5—C11'	76 (62)
C5—C6—C7—C8	−138.6 (2)	C12—C11—N5—C13	60.6 (19)
C1—C6—C7—C8	37.7 (3)	C12—C11—N5—C15'	−171.2 (15)
C5—C6—C7—C10	36.9 (3)	C16—C15—N5—C11	63 (2)
C1—C6—C7—C10	−146.7 (2)	C16—C15—N5—C11'	64 (2)
C10—C7—C8—O1	−179.2 (2)	C16—C15—N5—C13	−168.7 (16)
C6—C7—C8—O1	−3.6 (4)	C16—C15—N5—C15'	−95 (12)
C10—C7—C8—N3	−0.1 (3)	C12'—C11'—N5—C11	−118 (62)
C6—C7—C8—N3	175.5 (2)	C12'—C11'—N5—C15	171.4 (11)
C8—C7—C10—O3	−179.7 (2)	C12'—C11'—N5—C13	46.7 (8)
C6—C7—C10—O3	4.7 (4)	C12'—C11'—N5—C15'	174.3 (10)
C8—C7—C10—N4	−1.1 (3)	C14—C13—N5—C11	61.7 (10)
C6—C7—C10—N4	−176.7 (2)	C14—C13—N5—C15	−61.7 (11)
N5—C11—C12—O8	23 (3)	C14—C13—N5—C11'	61.4 (5)
N5—C15—C16—O9	45 (3)	C14—C13—N5—C15'	−69.2 (9)
C2—C3—N1—O4	156.9 (2)	C16'—C15'—N5—C11	66.1 (17)
C4—C3—N1—O4	−21.7 (3)	C16'—C15'—N5—C15	89 (11)
C2—C3—N1—O5	−23.3 (3)	C16'—C15'—N5—C11'	67.3 (14)
C4—C3—N1—O5	158.1 (2)	C16'—C15'—N5—C13	−161.9 (10)
C2—C1—N2—O7	−136.8 (2)		

Symmetry code: (i) −*x*+1, −*y*, −*z*+2.

### Hydrogen-bond geometry (Å, º)

**Table:**

*D*—H···*A*	*D*—H	H···*A*	*D*···*A*	*D*—H···*A*
O8—H8···O9^ii^	0.82	2.14	2.739 (10)	130
O9—H9···O11^iii^	0.82	2.11	2.841 (17)	148
N3—H3···O3^iv^	0.86	1.94	2.794 (2)	174
N4—H4*A*···O1^v^	0.86	1.97	2.804 (3)	165
N5—H5*A*···O10^iii^	0.97 (4)	1.86 (4)	2.808 (4)	164 (3)
O10—H10*B*···O2	0.90 (2)	1.87 (3)	2.751 (3)	163 (5)
O10—H10*A*···O6^v^	0.89 (2)	2.02 (2)	2.902 (4)	172 (6)

Symmetry codes: (ii) −*x*, −*y*+1, −*z*+1; (iii) *x*−1, −*y*+1/2, *z*−1/2; (iv) −*x*+1, *y*−1/2, −*z*+3/2; (v) −*x*+1, *y*+1/2, −*z*+3/2.
